# Disease-Course Adapting Machine Learning Prognostication Models in Elderly Patients Critically Ill With COVID-19: Multicenter Cohort Study With External Validation

**DOI:** 10.2196/32949

**Published:** 2022-03-31

**Authors:** Christian Jung, Behrooz Mamandipoor, Jesper Fjølner, Raphael Romano Bruno, Bernhard Wernly, Antonio Artigas, Bernardo Bollen Pinto, Joerg C Schefold, Georg Wolff, Malte Kelm, Michael Beil, Sigal Sviri, Peter V van Heerden, Wojciech Szczeklik, Miroslaw Czuczwar, Muhammed Elhadi, Michael Joannidis, Sandra Oeyen, Tilemachos Zafeiridis, Brian Marsh, Finn H Andersen, Rui Moreno, Maurizio Cecconi, Susannah Leaver, Dylan W De Lange, Bertrand Guidet, Hans Flaatten, Venet Osmani

**Affiliations:** 1 Division of Cardiology, Pulmonology and Vascular Medicine Medical Faculty, Heinrich-Heine-University Duesseldorf University Hospital Duesseldorf Duesseldorf Germany; 2 Fondazione Bruno Kessler Research Institute Trento Italy; 3 Department of Intensive Care Aarhus University Hospital Aarhus Denmark; 4 Department of Anaesthesiology Paracelsus Medical University Salzburg Austria; 5 Department of Intensive Care Medicine CIBER Enfermedades Respiratorias, Corporacion Sanitaria Universitaria Parc Tauli Autonomous University of Barcelona Sabadell Spain; 6 Department of Acute Medicine Geneva University Hospitals Geneva Switzerland; 7 Department of Intensive Care Medicine Inselspital, Universitätsspital University of Bern Bern Switzerland; 8 Department of Medical Intensive Care Hadassah University Medical Center, Faculty of Medicine Hebrew University of Jerusalem Jerusalem Israel; 9 Department of Anesthesia, Intensive Care and Pain Medicine Hadassah Medical Center and Faculty of Medicine Hebrew University of Jerusalem Jerusalem Israel; 10 Center for Intensive Care and Perioperative Medicine Jagiellonian University Medical College Krakow Poland; 11 Second Department of Anesthesiology and Intensive Care Medical University of Lublin Lublin Poland; 12 Faculty of Medicine University of Tripoli Tripoli Libyan Arab Jamahiriya; 13 Division of Intensive Care and Emergency Medicine Department of Internal Medicine Medical University Innsbruck Innsbruck Austria; 14 Department of Intensive Care 1K12IC Ghent University Hospital Ghent Belgium; 15 Intensive Care Unit General Hospital of Larissa Larissa Greece; 16 Mater Misericordiae University Hospital Dublin Ireland; 17 Department of Anaesthesia and Intensive Care Ålesund Hospital Alesund Norway; 18 Department of Circulation and Medical Imaging Norwegian University of Science and Technology Trondheim Norway; 19 Hospital de São José Centro Hospitalar Universitário de Lisboa Central Lisbon Portugal; 20 Faculdade de Ciências Médicas de Lisboa Nova Medical School - Faculdade de Ciências Médicas Universidade da Beira Interior Lisbon Portugal; 21 Department of Anaesthesia IRCCS Instituto Clínico Humanitas Humanitas University Milan Italy; 22 General Intensive Care St George's University Hospitals NHS Foundation Trust London United Kingdom; 23 Department of Intensive Care Medicine University Medical Center Utrecht University Utrecht Belgium; 24 Épidémiologie Hospitalière Qualité et Organisation des Soins Institut Pierre Louis d’Epidémiologie et de Santé Publique Sorbonne Universités, UPMC Univ Paris 06, INSERM, UMR_S 1136 Paris France; 25 Service de Réanimation Médicale Assistance Publique-Hôpitaux de Paris Hôpital Saint-Antoine Paris France; 26 Department of Clinical Medicine University of Bergen Bergen Norway; 27 Department of Anesthesia and Intensive Care Haukeland University Hospital Bergen Norway

**Keywords:** machine-based learning, outcome prediction, COVID-19, pandemic, machine learning, prediction models, clinical informatics, patient data, elderly population

## Abstract

**Background:**

The COVID-19 pandemic caused by SARS-CoV-2 is challenging health care systems globally. The disease disproportionately affects the elderly population, both in terms of disease severity and mortality risk.

**Objective:**

The aim of this study was to evaluate machine learning–based prognostication models for critically ill elderly COVID-19 patients, which dynamically incorporated multifaceted clinical information on evolution of the disease.

**Methods:**

This multicenter cohort study (COVIP study) obtained patient data from 151 intensive care units (ICUs) from 26 countries. Different models based on the Sequential Organ Failure Assessment (SOFA) score, logistic regression (LR), random forest (RF), and extreme gradient boosting (XGB) were derived as baseline models that included admission variables only. We subsequently included clinical events and time-to-event as additional variables to derive the final models using the same algorithms and compared their performance with that of the baseline group. Furthermore, we derived baseline and final models on a European patient cohort, which were externally validated on a non-European cohort that included Asian, African, and US patients.

**Results:**

In total, 1432 elderly (≥70 years old) COVID-19–positive patients admitted to an ICU were included for analysis. Of these, 809 (56.49%) patients survived up to 30 days after admission. The average length of stay was 21.6 (SD 18.2) days. Final models that incorporated clinical events and time-to-event information provided superior performance (area under the receiver operating characteristic curve of 0.81; 95% CI 0.804-0.811), with respect to both the baseline models that used admission variables only and conventional ICU prediction models (SOFA score, *P*<.001). The average precision increased from 0.65 (95% CI 0.650-0.655) to 0.77 (95% CI 0.759-0.770).

**Conclusions:**

Integrating important clinical events and time-to-event information led to a superior accuracy of 30-day mortality prediction compared with models based on the admission information and conventional ICU prediction models. This study shows that machine-learning models provide additional information and may support complex decision-making in critically ill elderly COVID-19 patients.

**Trial Registration:**

ClinicalTrials.gov NCT04321265; https://clinicaltrials.gov/ct2/show/NCT04321265

## Introduction

The COVID-19 pandemic caused by SARS-CoV-2 is continuing to challenge health care systems globally [1]. The disease disproportionately affects the elderly population, both in terms of disease severity and mortality risk [2]. In many countries, intensive care unit (ICU) capacity was increased during the pandemic to meet demand. In addition, novel treatment modalities were introduced [3]. A key challenge in clinical outcome prediction in a dynamic disease is that the response to a given treatment varies considerably from patient to patient, especially in the elderly population [4]. Baseline data alone are inadequate to predict prognosis with sufficient accuracy for an individual patient, as they cannot capture the dynamic nature of the underlying critical illness [5]. It is well established that various factors provide prognostic information that should be taken into consideration [6]. More elaborate methods are thus urgently needed for both sophisticated and concise risk stratification of severely affected individual ICU patients [7]. Biomarkers, frailty, and severity scores are validated in elderly critically ill patients [8-11]. However, all of these have important limitations as they do not reflect the dynamics of the underlying disease pathophysiology, and as a result have limited prognostic power. Ultimately, it remains up to the physician to integrate all baseline data, the changing course of the disease, and subjective experience into a clinical decision [12]. However, physicians do not assess dynamically evolving processes perfectly, as they are influenced by numerous factors, including fatigue and other human factors, resulting in less objective and reproducible decision-making [13]. This aspect is especially relevant for new diseases such as COVID-19, where physician experience is lacking.

Therefore, a supportive prognostication model that can integrate baseline data with complex, dynamic processes in an objective manner is necessary. Machine learning (ML) algorithms could be used to address this need, as some have successfully been evaluated in clinical settings such as in cardiovascular intensive care [14]. Wernly et al [9] retrospectively analyzed arterial blood gas data from septic intensive care patients from a multicenter electronic ICU database as well as from a single-center MIMIC-III (Medical Information Mart for Intensive Care) data set to predict 96-hour mortality.

Izquierdo et al [15] combined classical epidemiological methods, natural language processing, and ML to examine the electronic health records of 10,504 patients with COVID-19. According to their analysis, the combination of easily obtainable clinical variables such as age, fever, and tachypnea predicted which patients would require ICU admission [15]. The observational study by Bolourani et al [16] had a similar aim. They used clinical and laboratory data commonly collected in the emergency department to train and validate three predictive models (two based on extreme gradient boosting [XGB] and one that used logistic regression [LR]) with cross-hospital validation. The XGB model had the highest mean accuracy to predict 48-hour respiratory failure [16]. Aktar et al [17] used ML to distinguish between healthy people and those with COVID-19 and subsequently to predict COVID-19 severity. They used decision tree, random forest (RF), variants of gradient boosting machine, support vector machine, k-nearest neighbor, and deep learning methods for blood samples. The developed analytical methods evidenced accuracy and precision scores >90% for disease severity prediction. To avoid locally aggregating raw clinical data across multiple institutions, Vaid et al [18] evaluated a federated learning ML technique using electronic health records from 5 hospitals. In brief, they used LR with L1 regularization/least absolute shrinkage and selection operator, and multilayer perceptron models that were trained using local data at each study site. The federated models outperformed the local models with regard to their accuracy in predicting the mortality in hospitalized patients with COVID-19 within 7 days. In a smaller study, Domínguez-Olmedo et al [19] selected 32 predictor laboratory features in 1823 patients with confirmed COVID-19 for an XGB algorithm. Similar to the other studies, using laboratory parameters resulted in excellent outcome prediction. Subudhi et al [20] used ensemble-based ML models to identify C-reactive protein, lactate dehydrogenase, and oxygen saturation as the most important factors for predicting ICU admission, with estimated glomerular filtration rate <60 mL/min/1.73 m^2^, and neutrophil and lymphocyte percentages as the important factors for predicting mortality.

A recent systematic review by Syeda et al [21] identified more than 400 articles that investigated the role of ML in the field of COVID-19. For example, Pan et al [22] studied 123 ICU patients and identified eight important risk factors with high recognition ability using an XGB model. A similar approach was used by Kim et al [23], who established an XGB model in 4787 patients admitted to a hospital due to COVID-19. Furthermore, Burian et al [24] estimated the need for intensive care treatment in 65 patients with confirmed COVID-19, and Shahsikumar et al [25] investigated the performance of an algorithm to predict the need for mechanical ventilation on 402 patients with COVID-19, using cohorts with a wide age range (48 to 74 years).

Patients who are very old represent the most vulnerable intensive care subgroup [26]. However, to date, there are no studies investigating the role of ML models in this specific subgroup exclusively. To address this lack of evidence, the aim of this study was to evaluate whether ML models can reliably improve mortality prognostication in critically ill elderly patients with COVID-19 based on clinical baseline information, biomarkers, accumulating events, and time-to-event information during the disease course.

## Methods

### Study Design

This was a retrospective analysis that included data from 1432 patients in a prospective multicenter study. The primary outcome was 30-day mortality. We also used the 3-month outcome to ensure consistency of the primary outcome and allay concerns of censoring bias [27]. We derived two groups of models: baseline and final models. Baseline models were derived using admission variables only, whereas the final model group incorporated clinical events such as catecholamine therapy, renal replacement therapy, noninvasive ventilation, invasive ventilation, prone position, and tracheostomy, in addition to the baseline variables. We evaluated both model groups using stratified 3-fold cross-validation to mitigate the variability of a single derivation–validation random split. Furthermore, we derived baseline and final models on an EU patient cohort and externally validated them on a non-EU cohort that included Asian, African, and US patients.

### Clinical Data Sources and Study Population

Patient data were obtained from 151 ICUs across 26 independent countries, including European ICUs, and from ICUs in Asia, Africa, and the United States as part of the multinational COVIP trial (NCT04321265). This study was conducted in line with the European Union General Data Privacy Regulation directive. As in previous successful studies [6,26,28], national coordinators recruited the ICUs, coordinated national and local ethical permissions, and supervised patient recruitment at the national level. In the COVIP studies, ethical approval was obligatory for study participation. The electronic case report form (eCRF) and database were hosted on a secure server in Aarhus University, Denmark. Data from 1432 elderly (aged 70 years and above) COVID-19–positive patients admitted to a participating ICU between February 4 and May 26, 2020, were recorded. The study protocol is available from the COVIP study website [29]. Patients were followed up until hospital discharge and survival at 3 months using telephone interviews.

### Ethical Considerations

The primary competent ethics committee was the Ethics Committee of the University of Duesseldorf, Germany. Institutional research ethics board approval was obtained from each study site. This was a prerequisite for participation in the study. All methods were carried out in accordance with relevant guidelines and regulations. All experimental protocols were approved by the local institutional and/or licensing committees. Informed consent was obtained from all subjects if not omitted by the ethics vote. The studies were all observational; no examinations (eg, blood sampling) or tissue sampling took place.

### Study Data

Demographic data included age, gender, weight, height, and BMI. Furthermore, information on admission characteristics prior to ICU hospitalization, duration of hospital stay, day of symptom onset, and comorbidities were available. Preexisting comorbidities were recorded in the eCRF: diabetes, ischemic heart disease, renal insufficiency, arterial hypertension, pulmonary comorbidity, and chronic heart failure.

During the ICU stay, data on bacterial coinfection were noted, in addition to Sequential Organ Failure Assessment (SOFA) subscores (respiratory, cardiovascular, hepatic, coagulation, renal, and neurological systems). Laboratory values included partial oxygen pressure and the fraction of inspired oxygen (FiO2), and their ratio. Six clinical events of interest (catecholamine therapy, renal replacement therapy, noninvasive and invasive ventilation, prone position, and tracheostomy) were recorded along with the time the event occurred.

### Model Derivation and Validation

We derived models based on XGB [30], RF [31], and LR [32]. As the best-performing model, the XGB algorithm provides robust prediction results using a method where new models are added to correct the errors made by existing models. Models are added sequentially and the combination of many models in the XGB model accommodates nonlinearity between input variables [30]. Hyperparameter tuning was performed by an exhaustive grid search directed toward maximizing the F1-score metric. Three-fold cross-validation was performed inside each grid option, and the optimal hyperparameter set was chosen based on the model in the grid search with the highest F1 score. Hyperparameters of the final model of the XGB are listed in Multimedia Appendix 1. To generate confidence intervals for the baseline and the final models, 3-fold cross-validation was performed with 20-times repetition with a randomly generated seed. To compare the performance of the XGB model, we also derived and validated two more predictive models based on LR and RF. This decision was driven by the fact that LR is typically considered a baseline algorithm, and RF has been previously used in other research with COVID-19 data [33]. Both LR and RF were optimized by an exhaustive grid search, similar to the XGB method.

To address noise and outliers in the data, we defined a clinically valid interval for each variable, and the values out of the valid scope were considered as missing values. For all models, the issue of missing values was addressed by removing variables with >90% missing values. We then used the median and zero to impute the missing data in the remaining continuous and categorical variables, respectively. All analyses were carried out using open-source software based on Python 3.6.8 with scikit-learn version 0.23.2.

### Experimental Evaluation

**Figure 1:**
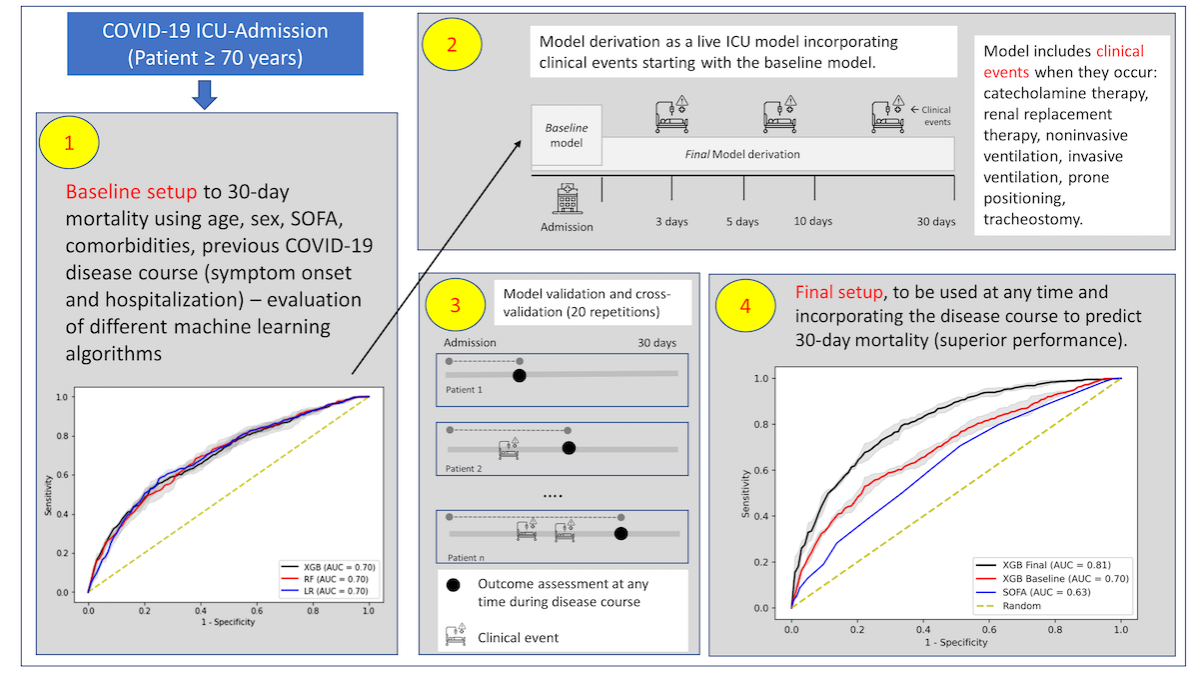
Graphical methods. (1) Study design, from admission to derivation and validation of baseline setup. (2) Derivation and validation of six models incorporating clinical events individually.Performance of individual models is shown in Multimedia Appendix 2-5. (3) Derivation of the final model, including baseline variables as well as clinical events. (4) Evaluation of the final model in predicting 30-day outcomes. SOFA: Sequential Organ Failure Assessment; ICU: intensive care unit.

Performance evaluation of the models was based on 3-fold, stratified cross-validation with 20 repetitions using the area under the receiver operating characteristic curve (AUC; see step 3 in Figure 1) as well as area under the precision-recall curve (PRC), also known as average precision [34].

The PRC shows the relationship between the positive predictive value (precision) and sensitivity (recall), measuring the performance of the model in correctly predicting mortality in patients with a high probability of dying. The area under the PRC is typically more informative than the AUC in the presence of imbalanced outcomes [34]. Additional performance metrics are detailed in Multimedia Appendix 2-5, including the positive predictive value (PPV), negative predictive value, F1 score (the balance between PPV and sensitivity), Matthews correlation coefficient (used to measure the quality of classification between algorithms), and Brier score. Calibration quality was evaluated using Brier scores, where a lower score indicates a higher calibration quality, and we also present calibration plots (also known as reliability curves). The models were compared based on their AUC and PRC performance metrics for both the baseline data as well as the final models incorporating clinical events.

### Model Interpretation

We evaluated the ranking of variables that contributed toward the model description using shapely additive explanation (SHAP) scores. SHAP scores are a game-theoretic approach to model interpretability; they provide explanations of global model structures based on combinations of several local explanations for each prediction [35]. To interpret and rank the significance of input variables toward the final prediction of the model, mean absolute SHA*P* values were calculated for each variable across all observations in both the baseline model and the final model based on XGB. We also plotted SHAP interaction values that capture the contribution of pairwise interactions between unique features to model prediction. To improve interpretability, especially in terms of the impact of clinical events, we defined a clinically meaningful day interval (0-3, 3-5, 5-10, and 10-30 days), and added a variable for each clinical event based on when the clinical event occurred; for example, “Tracheostomy-10-30” indicates that a tracheostomy was performed within the 10-30–day period. This allowed us to evaluate not only the importance of clinical events but also the time-to-event information. Naturally, these variables were only available in the final model.

## Results

### Study Population

Out of the total 1432 patients in the COVIP cohort, 809 (56.49%) patients survived up to 30 days after admission, with an average length of stay of 21.6 (SD 18.2) days. Patient baseline characteristics are given in Table 1, with distribution of mortality and length of stay detailed in Multimedia Appendix 6.

**Table 1:** Demographic characteristics, vital signs, and clinical events of patient cohorts (N=1432).

Variables		Alive at 30 days (n=809)	Dead at 30 days (n=623)	*P* value
Sex (male), n (%)		587 (72.6%)	463 (74.6%)	.18
Age (years), mean (SD)		75.0 (4.2)	76.5 (4.8)	<.001
Weight (kg), mean (SD)		81.3 (14.7)	81.0 (14.8)	.42
Height (cm), mean (SD)		169.7 (10.7)	169.8 (10.5)	.06
BMI (kg/m²), mean (SD)		28.5 (6.5)	28.4 (5.7)	.02
Hospital stay prior to ICU^a^ admission (days), mean (SD)		3.8 (5.7)	3.5 (6.3)	.002
Symptoms prior to hospital admission (days), mean (SD)		7.2 (5.2)	6.6 (4.5)	.10
PaO2^b^ (mmHg), mean (SD)		87.3 (44.2)	84.3 (57.5)	.003
FiO2^c^ (%), mean (SD)		62.3 (31.0)	73.0 (24.0)	<.001
SOFA^d^ score (points), mean (SD)		5.2 (3.0)	6.7 (3.4)	<.001
**ICU treatment and outcome**				
	Mechanical ventilation, n (%)	561 (69.3)	510 (81.9)	<.001
	Vasopressors, n (%)	525 (64.9)	515 (82.7)	<.001
	Prone positioning, n (%)	309 (38.2)	279 (44.8)	.10
	Tracheostomy, n (%)	227 (28.1)	64 (10.3)	<.001
	Noninvasive ventilation, n (%)	169 (20.9)	119 (19.1)	.32
	Renal replacement therapy, n (%)	121 (15.0)	119 (19.1)	.01
	Length of ICU stay (days), mean (SD)	21.6 (18.2)	10.6 (7.6)	<.001
**Preexisting comorbidities, n (%)**				
	Diabetes mellitus	268 (33.1)	240 (38.5)	.01
	Ischemic heart disease	151 (18.7)	152 (24.4)	.007
	Chronic renal insufficiency	91 (11.2)	130 (20.9)	<.001
	Arterial hypertension	527 (65.1)	431 (69.2)	.03
	Pulmonary disease	175 (21.6)	145 (23.3)	.07
	Chronic heart failure	98 (12.1)	103 (16.5)	.01

^a^ICU: intensive care unit.

^b^PaO2: partial oxygen pressure.

^c^FiO2: fraction of inspired oxygen.

^d^SOFA: Sequential Organ Failure Assessment.

### Model Derivation and Validation

We evaluated the performance of *baseline setup* risk prognostication that included baseline variables only (see step 1 in Figure 1) and the *final* setup, which—in addition to baseline variables—included six key clinical events that occurred during the disease course and their time-to-event information: catecholamine therapy, renal replacement therapy, noninvasive ventilation, invasive ventilation, prone positioning, and tracheostomy (step 2 in Figure 1). The final set of selected variables is shown in Table 1. Furthermore, the baseline and the final setup were used to derive models on the EU cohort of patients that were then externally evaluated using a non-EU cohort composed of Asian, African, and US patients.

Three risk prognostication models were derived from ML-based algorithms: LR and, for comparison, RF and XGB algorithms, as outlined in the Methods section [30,31].

**Figure 2:**
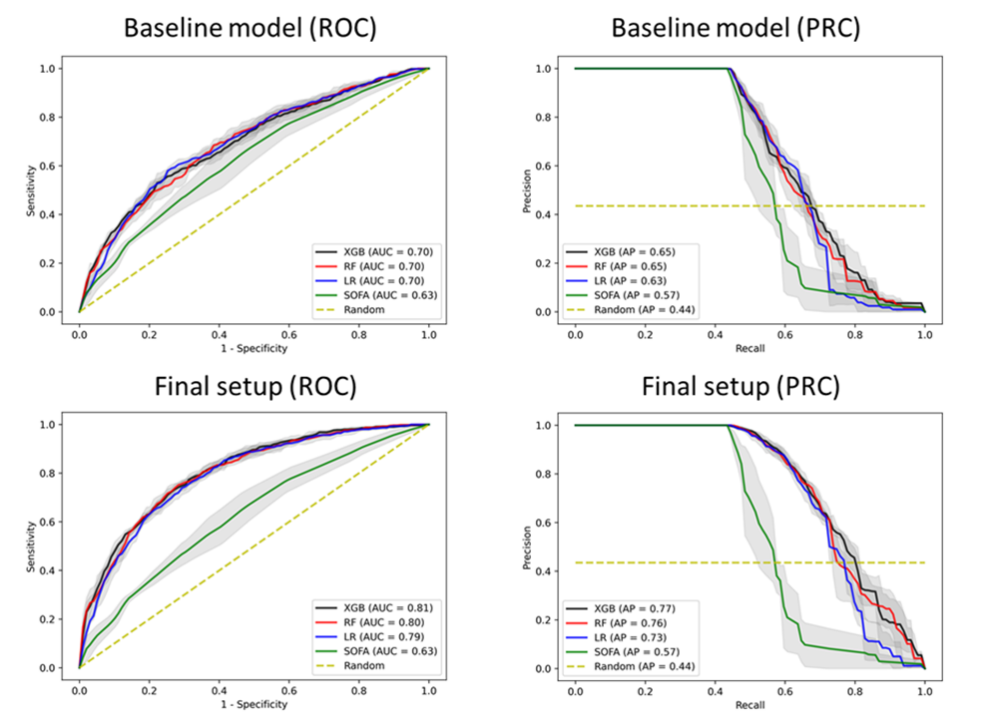
Performance of the baseline model (top) and improved performance in the final model (bottom) in response to clinical events with respect to the area under the receiver operating characteristic (ROC) curve (AUC) and area under the precision-recall curve (PRC). The PRC shows the relationship between the positive predictive value (precision) and sensitivity (recall) at all thresholds. XGB: extreme gradient boosting; RF: random forest; LR: logistic regression; SOFA: Sequential Organ Failure Assessment.

The XGB algorithm achieved the numerically highest increase in discrimination performance from the *baseline setup* (AUC 0.70, 95% CI 0.692-0.701) to the *final setup* (AUC 0.81, 95% CI 0.804-0.811); average precision increased from 0.65 (95% CI 0.650-0.655) to 0.77 (95% CI 0.759-0.770) (Figure 2).

The LR (AUC 0.79, 95% CI 0.788-0.796) and RF (AUC 0.80, 95% CI 0.798-0.805) algorithms showed similar performance in the *baseline model* and improvement in the *final model*, comparable to XGB performance (see step 4 in Figure 1). The final XGB model provided superior performance compared to both the baseline model and SOFA score (both *P*<.001).

### Experimental Evaluation

**Figure 3:**
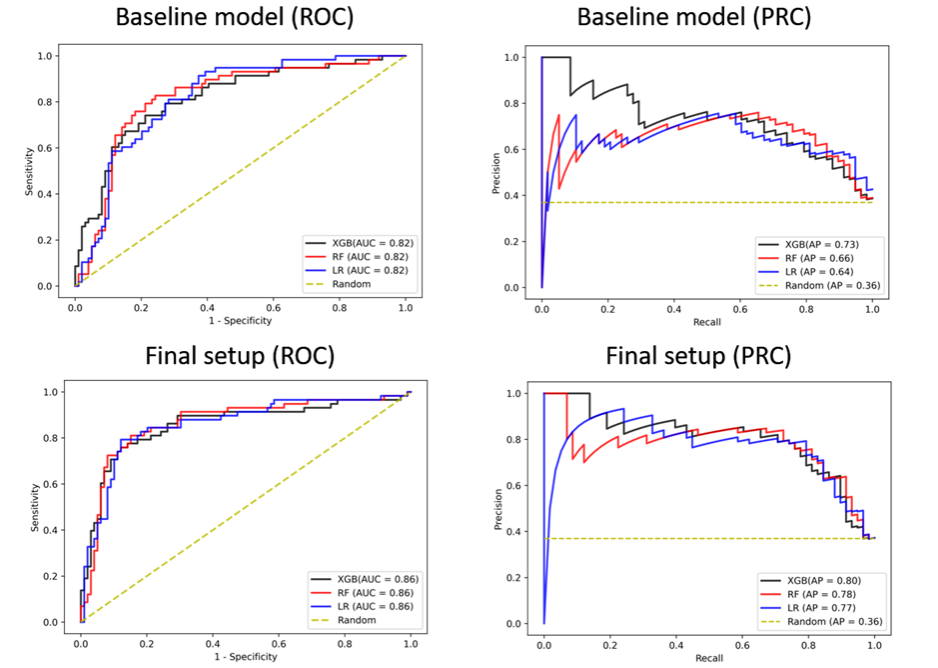
Performance of the final model derived using the EU patient cohort and externally validated on a non-EU patient cohort, comprising Asian, African, and US patients. Model performance is measured using area under the receiver operating characteristic (ROC) curve (AUC) and area under the precision-recall curve (PRC). XGB: extreme gradient boosting; RF: random forest; LR: logistic regression.

**Figure 4:**
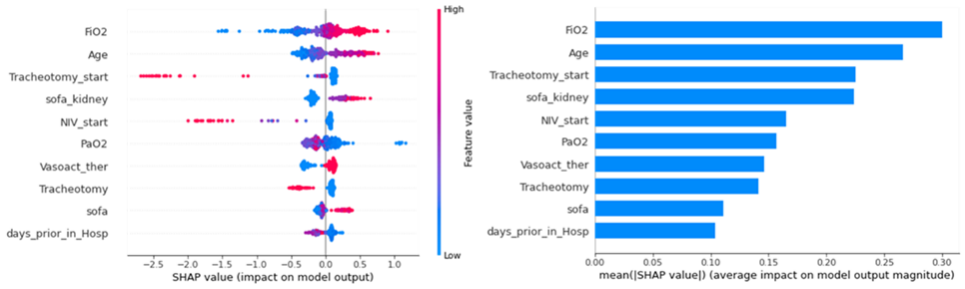
Ranking of input variables of the final setup derived from the extreme gradient boost algorithm, using the shapely additive explanation (SHAP) method.

In the external validation of the EU patient cohort, all three models achieved similar performance in the baseline and the final setup with an AUC of 0.82 and 0.86, respectively, when evaluated on predicting the mortality of non-EU patients (Figure 3). One explanation for this performance on the external validation cohort might be that the patients in the non-EU cohort tended to gravitate toward two opposing health states of either being quite stable or very sick, making it easier for the model to discriminate between the two outcomes. To investigate this further, we plotted the distribution of the variable that had the highest impact on outcome prediction (FiO2) based on SHAP analysis (see Figure 4). As shown in Multimedia Appendix 7, the distribution for both outcomes was significantly skewed toward 21% for survivors and toward 100% for nonsurvivors.

**Figure 5:**
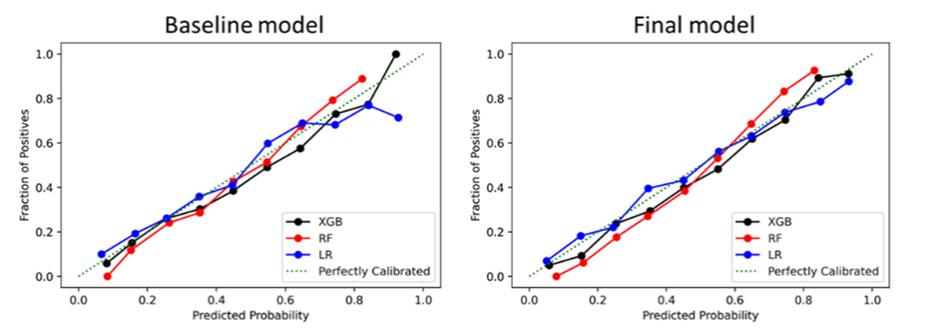
Calibration curves for each model and individual algorithms used to derive the model. XGB, extreme gradient boosting; RF: random forest; LR: logistic regression.

We also assessed the calibration of each model to ensure that the distribution of predicted outcomes matches the distribution of observed outcomes in our patient cohort. Baseline and final models were, in general, well calibrated (Figure 5), matching the estimated risk of outcome with observed risk. The final setup for each algorithm was better calibrated (Brier score of 0.17) with respect to the baseline setup (Brier score 0.22). Full details of Brier scores for each algorithm are detailed in Multimedia Appendix 1.

### Model Interpretation

The SHAP method was used to perform interpretability analysis, which explains model output by computing the contribution of each variable to the prediction. Among others, the SHAP method was applied on the best-performing model (XGB), where the FiO2, age, and tracheostomy had the highest impact on outcome prediction (Figure 4 and Multimedia Appendix 7).

We also report the model interpretability analysis for the RF- and LR-based models in Multimedia Appendix 8 and 9, respectively. The top three variables remained common between XGB and RF, whereas for LR, only tracheostomy appeared in the top three, with the other two high-ranking variables being weight and BMI.

## Discussion

### Principal Findings and Comparison With Related Studies

This study demonstrates that individual prognostication accuracy based on patient baseline characteristics can be considerably improved with ML algorithms that incorporate occurrence and time-to-event information of clinical events along the course of a disease such as COVID-19 in elderly, critically ill patients. These results align with many previous studies that investigated ML approaches in patients suffering from COVID-19. The major difference between this COVIP study and others published previously lies in its focus on the especially vulnerable subgroup of very old intensive care patients [21]. The second important difference is that the current approach includes the risk for clinical events such as tracheostomy.

Subudhi et al [20] compared the ability of 18 different ML algorithms to predict the rate of admission and mortality of patients suffering from COVID-19. In their analysis, ensemble-based models were superior to other algorithms (including LR and XGB). Specific laboratory values and oxygen saturation were the most important factors for ICU admission, whereas impaired kidney function and differential blood count best predicted mortality [20]. However, this previous study primarily used data from patients, of all ages, presenting to the emergency room.

Domínguez-Olmedo et al [19] used data from 1823 patients with confirmed COVID-19 and established an XGB model. Their model found lactate dehydrogenase activity, C-reactive protein level, neutrophil count, and urea level to be the most important variables, reaching an AUC of 0.93 (95% CI 0.89-0.98) for sensitivity and 0.91 (95% CI 0.86-0.96) for specificity.

Pan et al [22] used data from 123 patients with COVID-19 admitted to an ICU to construct an XGB model, and identified eight factors (albumin level, creatinine, eosinophil percentage, lactate dehydrogenase, lymphocyte percentage, neutrophil percentage, prothrombin time, and total bilirubin) that were predictive for ICU mortality.

Vaid et al [18] utilized a different approach based on federated learning of electronic health records from five different hospitals, providing robust predictive models without compromising patient privacy.

Other studies focused primarily on peripheral blood samples. Aktar et al [17] developed ML and deep learning algorithms to predict the disease severity. Similarly, Kim et al [23] established an XGB model in 4787 hospital-admitted patients to predict their intensive care treatment requirements. Their model was significantly superior to the established CURB-65 (confusion, urea, respiratory rate, blood pressure) score.

### Applications

Immediate clinical applications are conceivable, especially given the limited number of ICU beds available. Our models may be used in several ways: ML could be used before ICU admission to offer objective support for complex allocation decisions. However, ML algorithms would mainly access data at presentation and few dynamic parameters, limiting the predictive power. ML algorithms could also be used in the context of time-limited trials (TLTs), which are common clinical practice in ICUs in some countries. This may be particularly helpful in patients for whom realistic therapeutic goals/outcomes are unclear at presentation. These patients could be admitted to the ICU under the premise of gaining more information about the patient and the initial response to treatment. This additional information could then be evaluated using ML algorithms [36] as already shown in patients with sepsis [9]. The ideal temporal combination of a TLT and ML should be the subject of future, prospective studies [36,37].

In terms of practical applications, ML algorithms provide a potential strategy to improve decision confidence and predictive power over time. They are applicable at various time points during the disease course, predicting outcomes in a continuous manner. This approach is especially applicable when considering that the model was well calibrated in estimating outcomes. However, evaluation of the model with a diverse patient population would provide further evidence of its clinical applicability.

Clinical evaluations such as assessment of wakefulness, mobility, responsiveness, and independence are subjective and subject to interrater variability. Therefore, advances in digital technologies may support but not replace physicians’ skills. ML can support physicians, especially in estimations on prognosis and achievement of therapy goals. Importantly, ethical problems become evident when ML is involved in matters of life and death [38], and it must be emphasized that ML should only support and aid medical decision-making. Our data show that dedicated modern algorithms can incrementally improve certainty during TLTs in elderly patients with COVID-19, and generalize well in an external patient cohort. These tools can enhance our ability to improve guidance of treatment and optimally allocate ICU resources. However, such a strategy can only be viewed as complementary to clinical judgment and individual treatment goals, and form part of a holistic patient assessment.

### Limitations

This study has some methodological limitations in common with the other COVIP studies [11,26,39-42]. COVIP did not contain a control group of younger COVID-19 patients for comparison or a comparable age cohort of patients who were not or could not be admitted to the ICU. In addition, the COVIP database does not include information on pre-ICU care and triage decisions. These treatment limitations might also affect the care of older ICU patients [43]. Furthermore, COVIP recruited patients in 26 countries, and thus the participating countries varied widely in their care structure, resulting in considerable heterogeneity in treatments given.

### Conclusion

This study demonstrates that, in the particularly vulnerable subgroup of very old intensive care patients suffering from COVID-19, individual prognostication accuracy based on patient baseline characteristics can be improved with ML algorithms. These algorithms capture the dynamic course of the disease by including the occurrence and time-to-event information of clinical events, and thus reflect both disease severity and the need for intensive care treatment.

## Appendix

Multimedia Appendix 1 Hyperparameters for each algorithm found through an exhaustive grid search.

Multimedia Appendix 2 Performance of the baseline model in terms of various performance metrics and 95% CIs: logistic regression (LR), random forest (RF), extreme gradient boosting (XGB).

Multimedia Appendix 3 Performance of the final model in terms of various performance metrics and 95% CIs: logistic regression (LR), random forest (RF), extreme gradient boosting (XGB).

Multimedia Appendix 4 Performance of the baseline model derived using the EU patient cohort and validated using a non-EU patient cohort in terms of various performance metrics and 95% CIs: logistic regression (LR), random forest (RF), and extreme gradient boosting (XGB).

Multimedia Appendix 5 Performance of the final model derived using the EU patient cohort and validated using a non-EU patient cohort in terms of various performance metrics and 95% CIs: logistic regression (LR), random forest (RF), and extreme gradient boosting (XGB).

Multimedia Appendix 6 Distribution of deaths over time and length of intensive care unit stay.

Multimedia Appendix 7 Distribution of fraction of inspired oxygen (FiO2) for outcomes of survivors (left) and nonsurvivors (right). FiO2 was chosen as it was the variable that had the highest impact on the performance prediction, based on SHAP analysis.

Multimedia Appendix 8 Ranking of input variables of the final setup derived using the random forest–based model.

Multimedia Appendix 9 Ranking of input variables of the final setup derived using the logistic regression–based model.

Multimedia Appendix 10 List of COVIP-collaborators.

## Abbreviations

AUC: area under the receiver operating characteristic curve
CURB-65: confusion, urea, respiratory rate, blood pressure
eCRF: electronic case report form
FiO2: fraction of inspired oxygen
ICU: intensive care unit
LR: logistic regression
MIMIC-III: Medical Information Mart for Intensive Care
ML: machine learning
PPV: positive predictive value
PRC: precision-recall curve
RF: random forest
SHAP: shapely additive explanation
SOFA: Sequential Organ Failure Assessment
TLT: time-limited trials
XGB: extreme gradient boosting
