# Supplementary material for: Disease-Course Adapting Machine Learning Prognostication Models in Elderly Patients Critically Ill With COVID-19: Multicenter Cohort Study With External Validation
Source: JMIR Med Inform. 2022 Mar 31;10(3):e32949. doi: 10.2196/32949 (PMC9015783; doi:10.2196/32949)
Supplement: Multimedia Appendix 1 [file medinform_v10i3e32949_app1.docx]

| Multimedia Appendix 1:  Hyperparameters for each algorithm found through exhaustive grid search | | | | | | | |
| --- | --- | --- | --- | --- | --- | --- | --- |
| XGBoost | | | **Random Forest** | | | **Logistic Regression** | |
| Parameter | Baseline Model | Final model | **Parameter** | Baseline Model | Final model | **Parameter** | Both models |
| eta | 0.2 | 0.05 | estimators | 400 | 400 | penalty | l2 |
| max_depth | 2 | 3 | max_depth | 9 | 8 | solver | iblinear |
| min_child_weight | 1 | 1 | min_samples_split | 9 | 8 | class_weight | 0:1,1:1.2 |
| gamma | 0.2 | 0.3 | class_weight | 0:1,1:1.25 | 0:1,1:1.3 | C | 1.0 |
| colsample_bytree | 0.7 | 0.4 | criterion | gini | gini | - | - |
| scale_pos_weight | 1.25 | 1.25 | - | - | - | - | - |
