# Supplementary material for: Disease-Course Adapting Machine Learning Prognostication Models in Elderly Patients Critically Ill With COVID-19: Multicenter Cohort Study With External Validation
Source: JMIR Med Inform. 2022 Mar 31;10(3):e32949. doi: 10.2196/32949 (PMC9015783; doi:10.2196/32949)
Supplement: Multimedia Appendix 2 [file medinform_v10i3e32949_app2.docx]

| **Multimedia Appendix 2:**  Table showing the performance of the baseline model in terms of various performance metrics and 95% CI. | | | | | | | |
| --- | --- | --- | --- | --- | --- | --- | --- |
|  | **AUC** | **AP** | **PPV** | **NPV** | **MCC** | **F1** | **Brier** |
| **LR** | 0.70  [0.696- 0.701] | 0.63  [0.627- 0.633] | **0.65**  [0.651- 0.659] | 0.67  [0.671- 0.675] | **0.31**  [0.305- 0.318] | 0.56  [0.560- 0.568] | 0.22  [0.216- 0.218] |
| **RF** | 0.70  [0.692- 0.701] | 0.65  [0.638- 0.650] | 0.61  [0.601- 0.616] | 0.67  [0.668- 0.676] | 0.28  [0.265- 0.285] | 0.57  [0.563- 0.575] | 0.22  [0.216- 0.218] |
| **XGB** | 0.70  [0.692- 0.701] | **0.65**  [0.650- 0.655] | 0.61  [0.600- 0.606] | **0.69**  [0.688- 0.692] | 0.29  [0.287- 0.297] | **0.60**  [0.594- 0.601] | 0.22  [0.217- 0.219] |
| (AUC - area under the ROC curve; AP - average precision; PPV – positive predictive value; NPV – negative predictive value; MCC – Matthews correlation coefficient; F1 - harmonic mean of precision and recall and Brier score measuring quality of calibration, with lower values indicating better calibration). | | | | | | | |
