# Supplementary material for: Disease-Course Adapting Machine Learning Prognostication Models in Elderly Patients Critically Ill With COVID-19: Multicenter Cohort Study With External Validation
Source: JMIR Med Inform. 2022 Mar 31;10(3):e32949. doi: 10.2196/32949 (PMC9015783; doi:10.2196/32949)
Supplement: Multimedia Appendix 3 [file medinform_v10i3e32949_app3.docx]

| **Multimedia Appendix 3:**  Table showing the performance of the final model in terms of various performance metrics and 95% CI | | | | | | | |
| --- | --- | --- | --- | --- | --- | --- | --- |
|  | **AUC** | **AP** | **PPV** | **NPV** | **MCC** | **F1** | **Brier** |
| **LR** | 0.79  [0.788- 0.796] | 0.73  [0.721- 0.731] | **0.69**  [0.685- 0.694] | 0.75  [0.748- 0.755] | 0.44  [0.433- 0.444] | 0.68  [0.675- 0.682] | 0.18  [0.182- 0.186] |
| **RF** | 0.80  [0.798- 0.805] | 0.76  [0.748- 0.762] | 0.68  [0.676- 0.681] | 0.77  [0.766- 0.778] | 0.44  [0.446- 0.457] | 0.69  [0.690- 0.699] | 0.18  [0.182- 0.185] |
| **XGB** | **0.81**  [0.804- 0.811] | **0.77**  [0.759- 0.770] | 0.67  [0.668- 0.671] | **0.78**  [0.771- 0.783] | **0.45**  [0.443- 0455] | **0.70**  [0693- 0.703] | **0.17**  [0.176- 0.179] |
| (AUC - area under the ROC curve; AP - average precision; PPV – positive predictive value; NPV – negative predictive value; MCC – Matthews correlation coefficient; F1 - harmonic mean of precision and recall and Brier score measuring quality of calibration with lower values indicating better calibration). | | | | | | | |
