# Supplementary material for: Disease-Course Adapting Machine Learning Prognostication Models in Elderly Patients Critically Ill With COVID-19: Multicenter Cohort Study With External Validation
Source: JMIR Med Inform. 2022 Mar 31;10(3):e32949. doi: 10.2196/32949 (PMC9015783; doi:10.2196/32949)
Supplement: Multimedia Appendix 4 [file medinform_v10i3e32949_app4.docx]

| **Multimedia Appendix 4:**  Table showing performance of the baseline model derived using the E.U. patient cohort and validated using a non-EU patient cohort in terms of various performance metrics and 95% CI | | | | | | | |
| --- | --- | --- | --- | --- | --- | --- | --- |
|  | **AUC** | **AP** | **PPV** | **NPV** | **MCC** | **F1** | **Brier** |
| **LR** | 82 | 64 | 64 | 80 | 44 | 65 | **18** |
| **RF** | 82 | 66 | **71** | **83** | **54** | **71** | 19 |
| **XGB** | 82 | **73** | 69 | **83** | 51 | 70 | **18** |
| (AUC - area under the ROC curve; AP - average precision; PPV – positive predictive value; NPV – negative predictive value; MCC – Matthews correlation coefficient; F1 - harmonic mean of precision and recall and Brier score measuring quality of calibration, with lower values indicating better calibration). | | | | | | | |
