# Supplementary material for: Disease-Course Adapting Machine Learning Prognostication Models in Elderly Patients Critically Ill With COVID-19: Multicenter Cohort Study With External Validation
Source: JMIR Med Inform. 2022 Mar 31;10(3):e32949. doi: 10.2196/32949 (PMC9015783; doi:10.2196/32949)
Supplement: Multimedia Appendix 5 [file medinform_v10i3e32949_app5.docx]

| **Multimedia Appendix 5:**  Table showing the performance of the final model derived using the E.U. patient cohort and validated using a non-EU patient cohort in terms of various performance metrics and 95% CI (AUC - area under the ROC curve; AP - average precision; PPV – positive predictive value; NPV – negative predictive value; MCC – Matthews correlation coefficient; F1 - harmonic mean of precision and recall and Brier score measuring quality of calibration, with lower values indicating better calibration). | | | | | | | |
| --- | --- | --- | --- | --- | --- | --- | --- |
|  | **AUC** | **AP** | **PPV** | **NPV** | **MCC** | **F1** | **Brier** |
| **LR** | 86 | 77 | **79** | **87** | **66** | **78** | **15** |
| **RF** | 86 | 78 | 76 | 86 | 62 | 76 | 16 |
| **XGB** | 86 | **80** | 76 | **87** | 62 | 76 | **15** |
