# Supplementary figures and images for: Disease-Course Adapting Machine Learning Prognostication Models in Elderly Patients Critically Ill With COVID-19: Multicenter Cohort Study With External Validation
Source: JMIR Med Inform. 2022 Mar 31;10(3):e32949. doi: 10.2196/32949 (PMC9015783; doi:10.2196/32949)

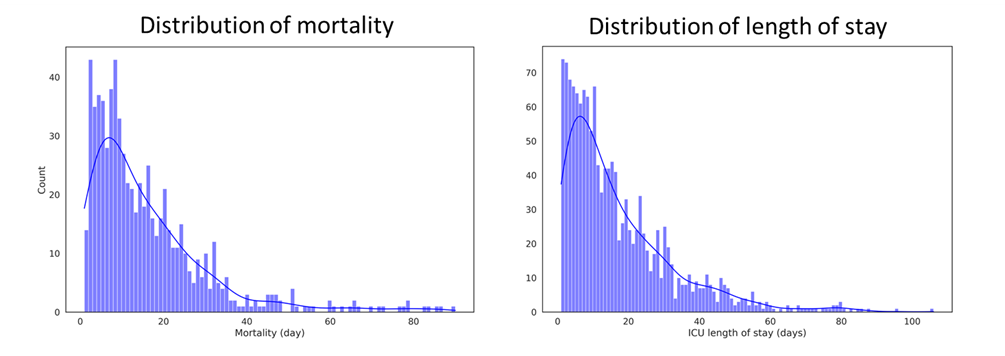

Supplement: Multimedia Appendix 6 [file medinform_v10i3e32949_app6.docx]

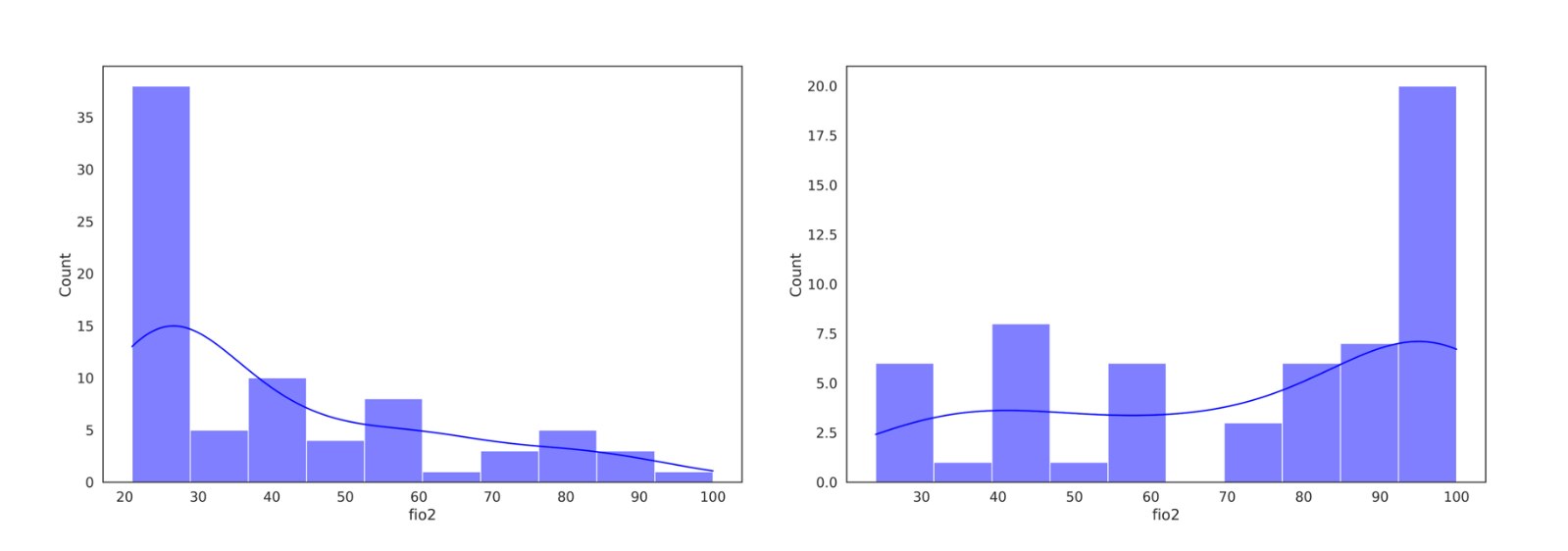

Supplement: Multimedia Appendix 7 [file medinform_v10i3e32949_app7.docx]

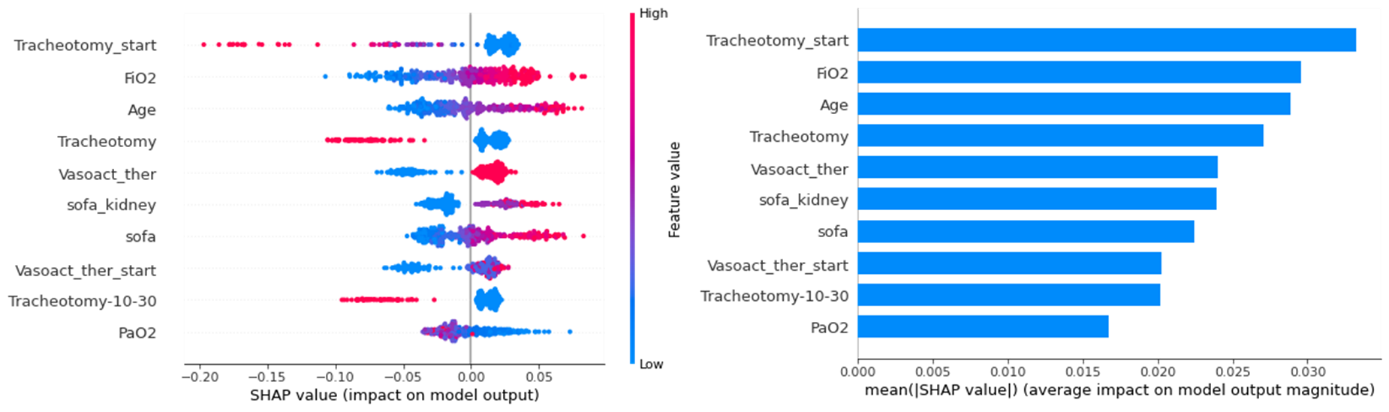

Supplement: Multimedia Appendix 8 [file medinform_v10i3e32949_app8.docx]

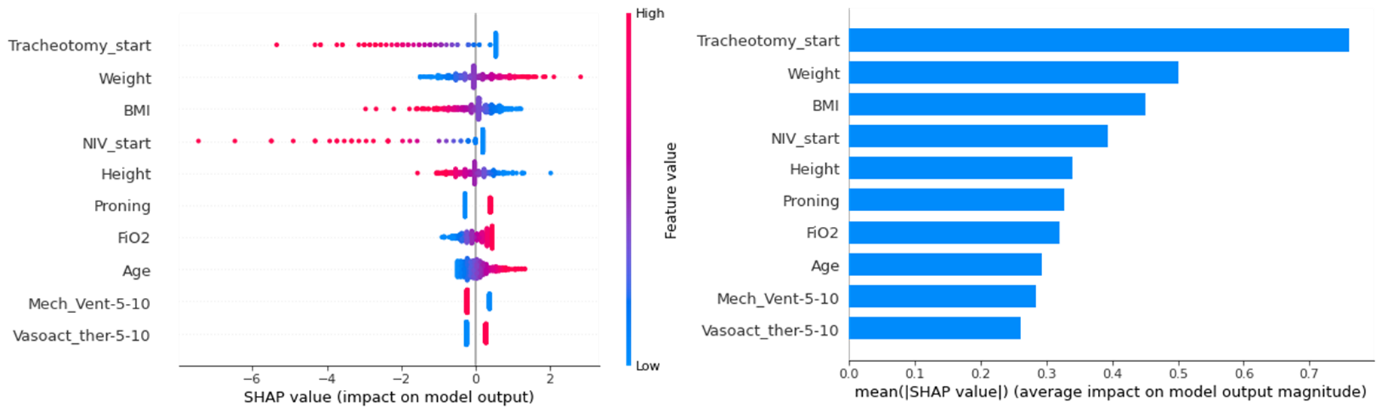

Supplement: Multimedia Appendix 9 [file medinform_v10i3e32949_app9.docx]
